# Impaired plasmacytoid dendritic cell maturation and differential chemotaxis in chronic hepatitis C virus: associations with antiviral treatment outcomes

**DOI:** 10.1136/gut.2008.168948

**Published:** 2009-02-04

**Authors:** J A Mengshol, L Golden-Mason, N Castelblanco, K A Im, S M Dillon, C C Wilson, H R Rosen, for the Virahep-C Study Group

**Affiliations:** 1 Division of Gastroenterology and Hepatology, Department of Medicine, University of Colorado Denver, Colorado, USA; 2 Integrated Program in Immunology and Hepatitis C Research Center, Denver, Colorado, USA; 3 Graduate School of Public Health, University of Pittsburgh, Pittsburgh, Phildelphia, USA; 4 Division of Infectious Diseases, Department of Medicine, University of Colorado, Denver, Colorado, USA; 5 Division of Clinical Immunology, Department of Medicine, University of Colorado, Denver, Colorado, USA

## Abstract

**Background:**

Dendritic cell (DC) defects may contribute to chronicity in hepatitis C virus (HCV) infection and determine response to PEG–interferon and ribavirin therapy via poor T cell stimulation. Studies to date have produced inconsistent results regarding DC maturation and function: no large study has examined DCs before and after therapy.

**Aims:**

We examined if DC defects in maturation and chemotaxis are present by comparing therapeutic responders to non-responders.

**Methods:**

We analysed peripheral DCs of 64 HCV genotype 1-infected patients from the Virahep-C study 2 weeks before and 24 weeks after therapy. We used flow cytometry to enumerate plasmacytoid DC (pDC) and myeloid DCs (mDC) and quantify expression of chemokine receptors and maturation markers. Chemotaxis was measured with an in vitro assay.

**Results:**

Pre-treatment frequencies of pDCs and mDCs were significantly lower in HCV patients than controls and successful therapy normalised pDCs. Levels of CXCR3 and CXCR4 on pDCs were higher at baseline compared to normal controls and decreased with therapy. Pre-therapy levels of co-stimulatory marker CD40 and the maturation marker CD83 were higher in pDCs of patients chronically infected with HCV compared to normal patients, and levels of both markers dropped significantly with therapy in the SVR+ group only. Other maturation markers (CD86 and CCR7) were not elevated suggesting a partially activated phenotype. Baseline chemotaxis of pDCs to CXCL12 and CXCL10 predicted failure of antiviral response and correlated with the histological activity index inflammation score.

**Conclusions:**

Plasmacytoid DC defects exist in chronic HCV and successful antiviral therapy normalises many phenotypic and functional abnormalities.

Hepatitis C virus (HCV) has a global prevalence of 3% and up to 70% of individuals exposed to HCV develop viral persistence.^1^ ^2^ HCV causes substantial worldwide morbidity and mortality. Twenty per cent of infected patients eventually develop cirrhosis and HCV infection is a leading cause of liver transplantation.^3^ Current therapy with interferon and ribavirin is only effective in about half of patients and causes significant morbidity.^4^ Thus understanding the mechanism of therapeutic success and failure has important clinical relevance for developing improved therapies and predicting non-response. Patients who spontaneously clear infection have strong and broad T cell responses while patients with chronic HCV have weak and functionally impaired responses characterised by poor proliferation, impaired cytotoxicity and reduced cytokine secretion after antigen exposure.^3^ ^5^^–^7 Higher pre-treatment CD4^+^ T cell interferon γ responses to HCV proteins are associated with higher rates of sustained virological response (SVR).^8^

Dendritic cells (DCs) are efficient and potent antigen presenters and activators of antigen-specific T cells and adaptive immunity.^9^^–^12 Defective DC activation of T cells may underlie poor T cell responsiveness in HCV infection, and may, in part, determine the response to therapy.^13^ Human peripheral blood DCs are currently categorised into two major subsets: myeloid DCs (mDCs) and plasmacytoid DCs (pDCs). Myeloid DCs are effective antigen presenters to T cells and secrete interleukin 12, while pDCs are the most potent secretors of antiviral type-I interferons like interferon α.^10^ DCs migrate to sites of inflammation, sample antigens, and integrate generic microbial danger signals via innate immune receptors, named pathogen recognition receptors (PRRs), that recognise pathogen-associated molecular patterns (PAMPs).^13^ PRR signals combine with signals from inflammatory cytokines to activate DCs, causing up-regulation of co-stimulatory molecules like CD40 and CD86. DCs then migrate to lymphoid tissue where they activate antigen-specific CD4 and CD8 T cells by presenting antigens on major histocompatibility complex (MHC) class I and II molecules.^11^ ^14^

Reports of global immune dysfunction in HCV infection are controversial; some studies have suggested that HCV patients have poorer response to hepatitis B vaccination^15^ and higher rates of infection with herpes simplex virus (HSV).^16^ However, strong global immune dysfunction as seen in HIV/AIDS is not seen. DC dysfunction may be restricted to the HCV-specific response. Studies of impaired DC function in chronic HCV have yielded variable results; most use monocyte-derived DCs from HCV patients, not DCs analysed directly ex vivo. Some authors have found faulty responses to general PRR stimulation including decreased IFNα and IL12 secretion, reduced CD86 expression, decreased HLA-DR (MHC class II) and impaired stimulation of T cells in mixed lymphocyte reaction compared with normal controls.^13^ ^17^^–^24 Specific HCV proteins like core and E2 can cause DC dysfunction in tissue culture models.^13^ ^19^ Other authors, including those using direct ex vivo human samples or a chimpanzee model of HCV have found no defects.^13^ ^25^^–^29 It has been consistently shown in HCV infection that pDC and mDC numbers are reduced in the peripheral compartment compared with normal controls, whereas reports have described increased numbers of DCs in the livers of HCV patients, suggesting hepatic DC sequestration.^13^ ^18^ ^22^ ^30^ ^31^ The unresolved controversies listed above highlight the need for further study of DCs in HCV infection.

Chemokines and chemokine receptors are important regulators of DC migration to specific anatomic areas such as lymph nodes and sites of infection, and thus have an important effect on DC function. Chemokines are elevated in HCV patients,^32^ and in vitro studies have suggested that the HCV protein E2 can inhibit DC chemotaxis.^30^ Serum levels of the chemokines IL8 (CXCL8) and IP-10 (CXCL10) are elevated compared with normal controls and increased levels are associated with higher HCV viral levels and disease activity while lower levels have been associated with improved response to combination therapy.^33^^–^35 Additionally, HCV patients have high levels of CXCR3, CXCR4, CCR5 and CCR7 expression on liver-infiltrating lymphocytes, and the corresponding chemokines for these receptors are also increased in the liver and the plasma.^32^ ^36^ To date, no large study has examined levels of these receptors on DC subsets or chemotaxis of DC before and after therapy for HCV directly ex vivo.

We hypothesised that baseline differences in DC maturation and function may determine an individual’s response to PEG–IFN/ribavirin therapy. We also postulated that patients who ultimately have a sustained virological response (SVR) might show temporal differences in DC maturation and function with therapy. In contrast to prior studies of DC maturation in HCV which used cultured-derived DCs, we studied patient samples analysed directly ex vivo from the large Virahep-C treatment trial. We examined chemokine receptors, maturation markers, and co-stimulatory markers on DC subsets, as well as the ability of pDCs and mDCs to migrate to chemokines relative to histological injury and treatment outcome. For the first time, we demonstrate incomplete maturation of the pDC subtype in hepatitis C, which resolves with successful therapy. We also show that therapeutic non-responders have increased pDC migration to inflammatory chemokines before therapy when compared with therapeutic responders.

## METHODS

### Study population

Virahep-C is a multicentre collaborative observational study primarily designed to examine racial differences (Caucasian vs African–American) in naïve HCV genotype 1 treatment response rates to combination therapy (pegylated IFNα-2a and ribavirin). The methods and overall demographics of the 401 patients in the Virahep-C study have been described previously.^37^ Patients received 48 weeks of combination therapy unless they were HCV RNA positive at week 24; in these patients, therapy was stopped. We randomly selected a cohort of 64 patients with adequate cell numbers from the Virahep-C group based on matching equal numbers of patients in four groups by sustained virological response (SVR defined as HCV RNA negative 24 weeks after the end of therapy) and race (Caucasian vs African–American). Extensive demographic data was available on all patients and the characteristics of the study cohort are shown in table 1. The data in our cohort did not differ significantly from the full cohort of 401 patients.^37^ All patients were genotype-1, the mean age was 48 years; 64% were male and 53% were African–American (AA). Ninety seven per cent of patients had an Ishak score 4/6 or lower and the mean histological activity index (HAI) score was 8.6/18. Seventy per cent of patients had an early virological response (greater than 2 log decrease in HCV RNA after 12 weeks of therapy). Thirty patients achieved SVR while 34 did not. SVR positive patients were more likely to be female, have lower levels of insulin resistance (homeostasis model assessment (HOMA)), and have an early virological response (EVR) and rapid virological response (RVR) as described previously and shown in table 1.^37^

**Table 1:** Demographic and clinical features of 64 patients from the Virahep-C study

	n = 64	Sustained virological response	
		Non-responders	Responders
		(n = 34)	(n = 30)
Age (years)			
Mean (SD)	48.1 (8.4)	48.1 (9.4)	48.1 (7.3)
Median (25th, 75th)	48 (44.0, 53.0)	48 (45.0, 54.0)	48 (43.0, 50.0)
Race			
African–American	34 (53.1%)	19 (55.9%)	15 (50.0%)
Caucasian–American	30 (46.9%)	15 (44.1%)	15 (50.0%)
Gender			
Male	41 (64.1%)	26 (76.5%)	15 (36.6%)
Female	23 (35.9%)	8 (34.8%)	15 (65.2%)
Genotype			
1a	34 (53.1%)	19 (55.9%)	15 (50.0%)
1b	24 (37.5%)	15 (44.1%) 0 (0%)	9 (5.3%)
Other	6 (9.4%)		6 (0.2%)
1	3		3
1a/b	2		2
Indeterminate	1		1
Viral level, IU/ml (log_10)_			
Mean (SD)	6.3 (0.7)	6.4 (0.7)	6.1 (0.6)
Median (25th, 75th)	6.4 (5.7, 6.8)	6.5 (6.2, 6.9)	6.2 (5.6, 6.5)
Alanine aminotransferase (IU/l)			
Mean (SD)	84.8 (77.3)	83.2 (57.2)	86.6 (96.3)
Median (25th, 75th)	60.0 (41.5, 99.5)	65.5 (50.0, 105.0)	53.0 (38.0, 98.0)
Total HAI inflammation			
Mean (SD)	8.6 (2.9)	8.8 (2.8)	8.3 (3.1)
Median (25th, 75th)	8.0 (6.5, 11.0)	8.0 (7.0,11.0)	8.0 (6.0, 11.0)
Ishak fibrosis score			
0	12 (18.8%)	4 (11.8%)	8 (26.7%)
1, 2	29 (45.3%)	15 (44.1%)	14 (46.7%)
3, 4	21 (32.8%)	13 (38.2%)	8 (26.7%)
5, 6	2 (3.1%)	2 (5.9%)	0 (0.0%)
HOMA			
⩽2	30 (46.9%)	13 (38.2%)	17 (56.7%)
>2	25 (39.1%)	15 (44.1%)	10 (33.3%)
Missing	9 (14.1%)	6 (17.7%)	3 (10.0%)
28 day response			
Poor	37 (57.8%)	26 (76.5%)	11 (29.7%)
Intermediate	10 (15.6%)	3 (8.8%)	7 (23.3%)
Marked	16 (25.0%)	4 (11.8%)	12 (40.0%)
Rebound >1.4	1 (1.6%)	1 (2.9%)	0 (0.0%)
Early virological response (week 12 response)			
Non-responder	19 (29.7%)	18 (52.9%)	1 (3.33%)
Responder	45 (70.3%)	16 (47.1%)	29 (96.7%)

At 28 days poor response was defined as </ = 1 log decrease, intermediate 1–3.5 log decrease, marked >3.5 log decrease in viral level. Early virological response was defined as RNA negativity at 12 weeks of therapy. Sustained virological response was defined as RNA negativity at 24 weeks after cessation of therapy.

HAI, histological activity index; HOMA, homeostasis model assessement.

PBMCs from 20 healthy volunteer patients served as normal controls. The average age of controls was 37; 63% were male, and 50% were AA.

PBMCs were isolated from whole blood by Ficoll (Amersham, Piscataway, New Jersey, USA) density gradient centrifugation or cellular preparation tubes (Becton-Dickinson, San Jose, California, USA). PBMCs were viably frozen in 80% fetal bovine serum (BioWhittaker, Basel, Switzerland), 10% dimethyl sulfoxide (DMSO), and 10% RPMI 1640 media (Life Technologies, Carlsbad, California, USA) in liquid nitrogen for subsequent analyses.

### Flow cytometry

PBMCs were thawed into RPMI with 10% human serum, and two million PBMCs per tube were stained with the following antibodies according to the manufacturer’s recommendations. BDCA-1,2 and FcR blocking reagent were from Miltenyi (Bergisch Gladbach, Germany). CXCR3, CXCR4, CCR5, CCR7, CD83, CD86, DC-sign were from BD biosciences (Becton Dickinson). CD40 was from Ancell (Bayport, Minnesota, USA). Appropriate isotype control antibodies were utilised. After incubation with antibodies for 20 min in the dark at 4°C, cells were washed once with cold phosphate-buffered saline (PBS) containing 0.6% bovine serum albumin and 0.01% sodium azide and fixed in 2.5% paraformaldehyde. Acquisition and analysis was performed using a Becton-Dickinson FACSCalibur flow cytometer running CellQuest Pro software at the University of Colorado Denver Clinimmune flow cytometry core facility. Because BDCA-1 is expressed on a subset of CD19^+^ B-cells, we tested for CD19^+^ cells in the BDCA-1 gate. We found gating on high BDCA-1 expression reduced CD19^+^ cells to less than 5% of the mDC gate (data not shown). Some patients did not have enough PBMCs saved to analyse every marker therefore small variations occurred in patient numbers per experiment.

### Chemotaxis

Twenty patients from the original cohort of 64 patients were chosen for study based on the availability of sufficient numbers of cells for analysis. Seven normal patients from the group mentioned above were used as controls. CXCL12 and CXCL10 chemokines were purchased from Preprotech. Two million PBMCs in RPMI in 10% human serum were added to the upper well of a 6.5 mm, 5 μm pore transwell chamber (Corning, Pittsburgh, Philadelphia, USA).^38^ The bottom chamber contained the same media plus chemokine. After a 3 h incubation, cells in the bottom chamber were collected using ice cold PBS containing 10 mmol/l EDTA. The collected cells were stained with BDCA-1–APC and BDCA-2–FITC and counted in a BD Canto flow cytometer. Fifty thousand unlabelled beads (Bang Laboratories, Fishers, Indiana, USA) were added to each sample and used as a control for counting efficiency. We performed each analysis in duplicate. We calculated and plotted the chemotaxis index, defined as the ratio of cells migrated with chemokine divided by the number of cells migrated without chemokine.

### Statistical analysis

Descriptive statistics, including medians, means, standard deviations and frequencies are tabulated for the raw counts separately for the patient groups. The two-tailed non-parametric Wilcoxon signed rank-sum test was used to compare paired DC measurements before and after therapy. Correlation between chemotaxis and histological activity index were assessed using the Spearman rank correlation coefficient. In order to examine the association between baseline DC measurements and SVR, weighted generalised estimating equations (GEEs) modelling approach (proc genmod in SAS) was utilised. Comparisons in DC measurements between the HCV cohort and normal controls were done by permutation test using a sum of ranks as a statistic since we have relatively unbalanced sample sizes between the two groups. A p value of less than 0.05 was considered significant and not adjusted for multiple testing. Permutation tests were done in R package version 2.7 (available at http://CRAN.R-project.org/). Other statistical analyses were performed using JMP 6.0, SAS 9.1.3 and Prism 5.01 (GraphPad Software, San Diego California USA).

## RESULTS

### Flow cytometry

We analysed PBMCs that were isolated 2 weeks before initiation (screening 2 weeks, “S002”) and 24 weeks after cessation of therapy (follow-up week 24, “FU24”) by flow cytometry. Figure 1 demonstrates the gating strategy utilised to separate and analyse plasmacytoid dendritic cells (pDCs) and myeloid dendritic cells (mDCs). As previously described by other groups, mDCs were defined as BDCA-1 positive and pDCs were defined as BDCA-2 positive.^30^ ^39^^–^43

**Figure 1:**
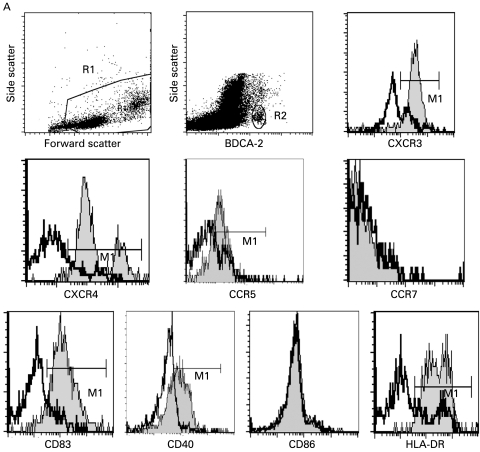
Representative gating strategy for isolation of dendritic cell (DC) subtypes. Two million PBMCs were stained with BDCA-1-APC and BDCA-2-FITC. Additionally, peridinin chlorophyll protein (PerCP) and R-phycoerythrin (PE)-labelled antibodies were used to measure surface marker expression by 4-color flow cytometry. Forward scatter and side scatter identified and gated live cells in region 1 (R1) (A,B). Side scatter and either BDCA-2 (A, pDC) or BDCA-1 (B, mDC) were used to identify and gate DC subtypes in region 2 (R2).^39^ ^40^ Overlay plots comparing isotype antibody controls with specific marker staining were used. The thicker line is the isotype control, the thinner line shaded with grey is staining with antibody to the protein indicated below the graph. The gate used is the horizontal bar labelled M1. (A) pDC gating and representative expression. APC, allophycocyanin; FITC, fluorescein isothiocyanate; mDCs, myeloid dendritic cells; pDCs, plamscytoid dendritic cells; PMBCs, peripheral blood mononuclear cells. (*Figure 1B** is on p 967.*)

### Plasmacytoid and myeloid dendritic cells are decreased in chronic HCV

Consistent with prior studies,^18^ ^19^ ^44^ ^45^ we found the percentage of pDCs in chronic HCV patients (median 0.32%, range 0.08–1.44%) was slightly reduced compared with normal patients (0.39%, 0.17–5.1% p = 0.0323) (fig 2). Chronic HCV patients had slightly lower levels of mDCs (median 0.53%, range 0.16–2.08%) compared with normal patients (0.73%, 0.33–1.56%, p = 0.0245) (fig 2). In SVR+ patients (fig 3A), the per cent of pDCs increased significantly towards normal controls after therapy (median 0.31–0.36%, p = 0.0497), while in the SVR− patients, they did not (0.33–0.30%). In contrast, mDCs did not change significantly in either group (fig 3A). No significant pre-treatment differences in per cent pDCs or mDCs was noted comparing SVR+ and SVR− patients.

**Figure 1 Continued:**
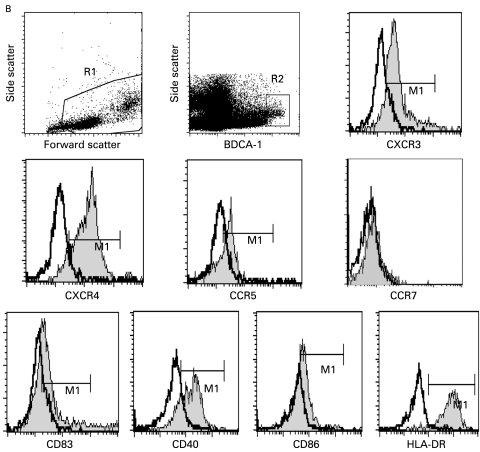
Representative gating strategy for isolation of DC subtypes. Two million PBMCs were stained with BDCA-1–APC and BDCA-2–FITC. Additionally, PerCP and PE labelled antibodies were used to measure surface marker expression by four-colour flow cytometry. Forward scatter and side scatter identified and gated live cells in region 1 (R1) panels (A,B). Side scatter and either BDCA-2 (A, pDC) or BDCA-1 (B, mDC) were used to identify and gate DC subtypes in region 2 (R2).^39^ ^40^ Overlay plots comparing isotype antibody controls with specific marker staining were used. The thicker line is the isotype control, the thinner line shaded with grey is staining with antibody to the protein indicated below the graph. The gate used is the horizontal bar labelled M1. (B) mDC gating and representative expression. Median fluorescence intensity in the gated region M1 was calculated by CellQuest Pro software and used in subsequent analysis.

**Figure 2:**
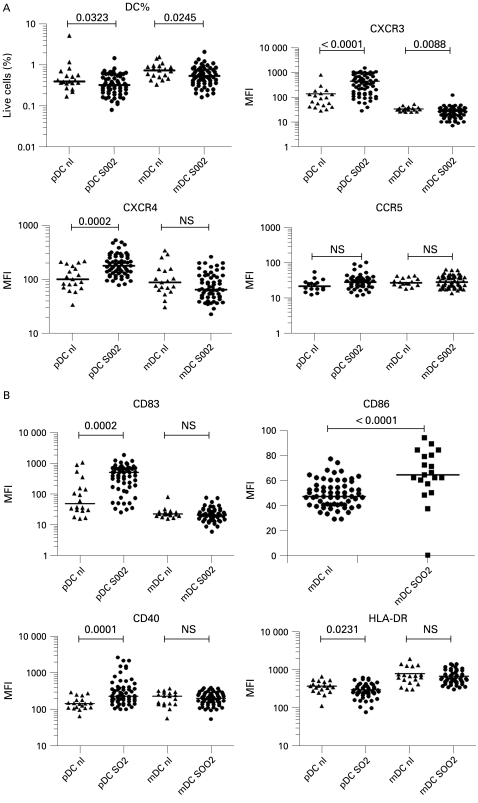
Comparison of plasmacytoid dendritic cells (pDCs) and myeloid dendritic cells (mDCs) between normal controls and hepatitis C virus (HCV) patients 2 weeks before starting therapy. SOO2 is the visit 2 weeks before initiation of therapy. FU24 is 24 weeks after cessation of treatment. Median fluorescence intensity (MFI) values for each patient are shown. The horizontal line is the median. A two-tailed Mann–Whitney test was used to calculate p values. (A) Per cent of cells in region R1 is shown, CXCR3 MFI, CXCR4 MFI and CCR5 MFI. (B) CD83, CD86, CD40, HLA-DR MFI.

### Inflammatory chemokine receptors increased on plasmacytoid dendritic cells in HCV patients

We measured levels of DC chemokine receptors before and after therapy, focusing on the inflammation-homing receptors CXCR4, CXCR3, CCR5 and CCR7, the latter being important for mature DCs to migrate to lymphoid organs to present antigen.^30^ ^33^ ^34^ ^36^ Plasmacytoid DC MFI levels of CXCR4 (median 181, range 79–542) and CXCR3 (median 362, range 28–1582) were significantly elevated before therapy compared with normal controls (median 102, range 34–219, p = 0.0002, median 90, range 28–820, p<0.0001, respectively) (fig 2A). Combination therapy significantly decreased pDC CXCR4 expression toward normal levels with a larger decrease seen in patients with SVR compared with non-SVR patients (median 193 to 115 compared with median 177 to 160, fig 3A). CXCR3 levels on pDCs also decreased significantly after therapy in both SVR+ (p = 0.0001) and SVR− (p = 0.0018) patients (fig 3A). In mDCs, CXCR4 baseline levels were not elevated compared with normal controls and a decrease in expression levels occurred in SVR+ patients only (p = 0.0004) (figs 2A, 3A). Levels of CXCR3 on mDCs were decreased compared with normal controls and decreased with successful therapy only (figs 2A, 3A). CCR5 expression levels were not significantly different on pDCs and mDCs between normal subjects (fig 2A). CCR7 expression was either extremely low or undetectable on pDCs and mDCs (fig 1A,B) consistent with prior reports of low peripheral expression levels in unstimulated pDCs.^25^ ^46^ ^47^

### HCV plasmacytoid dendritic cells have a partially activated phenotype

We also measured levels of the co-stimulatory molecules CD40 and CD86, MHC class II (HLA-DR), the maturation marker CD83, and the viral binding protein DC-SIGN. We observed that pre-treatment pDCs from patients had elevated levels of CD40 (median 234, range 102–2641) and CD83 (median 514, range 26–1860) compared with normal controls (147, 66–294, p = 0.0001; 50, 15–1065, p = 0.0002) (fig 2B). A significant decrease in CD40 and CD83 towards normal values occurred only in SVR+ patients (p = 0.0365, p = 0.0068, respectively, fig 3B) compared with SVR− patients whose levels remained elevated. Pre-treatment HLA-DR levels (median 275, range 79–635) were significantly lower on pDCs compared with normal controls (median 375, range 115–679) while mDCs were not significantly different (fig 2). SVR− patients had a small but statistically significant decrease in mDC HLA-DR MFI (p = 0.0102) after therapy while SVR+ patients did not (fig 3B). CD86 levels on mDCs were decreased compared with normal patients and no significant change in MFI occurred after therapy. CD86 expression was generally low to absent on pDCs from all groups (no increase above isotype control, fig 1A). CD83 mDC levels were not significantly different from controls in all groups at all time points (fig 2 and data not shown). DC-SIGN was not detected on pDCs and mDCs in PBMC from all groups (data not shown).

### No association between pre-treatment expression levels of dendritic cell markers and sustained virological response

The associations between pre-treatment DC measurements described above and SVR were examined by weighted GEE-type regression analysis adjusted for race. In addition, we also examined a possible confounding effect of other clinical and laboratory characteristics of the cohort by including a subject’s gender, age, baseline histology using HAI scores, baseline viral levels, ALT level, and measurement of insulin resistance (HOMA) one at a time in each regression model. Overall, we did not observe significant association between DC markers and chemokines and SVR whether the model was adjusted or unadjusted for clinical and laboratory characteristics (data not shown).

### Baseline chemotaxis is higher in patients with sustained viral release: correlation of dendritic cell chemotaxis to hepatic inflammation

Since chemotaxis has such an important role in DC function, we next examined whether differences in chemokine surface receptor expression led to functional differences in DC chemotaxis. Because CXCR4 and CXCR3 chemokine receptors levels were elevated on pDC, and these levels decreased more in patients who had SVR, we decided to analyse the ability of DCs to migrate towards chemokines SDF-1 (CXCL12) and IP-10 (CXCL10), which signal through CXCR4 and CXCR3, respectively.^32^ We selected 20 patients (10 SVR+, 10 SVR−) with sufficient PBMCs for analysis from the original cohort of patients before and after HCV therapy. Overall, there was greater pDC and mDC migration towards CXCL12 than CXCL10 in both normal patients and chronic patients before therapy (fig 4). Combining all patients at timepoint S002, the mean chemotaxis index of pDC to CXCL12 was 4.708 versus 1.007 for CXCL10 (p<0.0001). Mean migration for mDCs was 6.533 to CXCL12 vs 1.255 to CXCL10 (p<0.0001).

**Figure 3:**
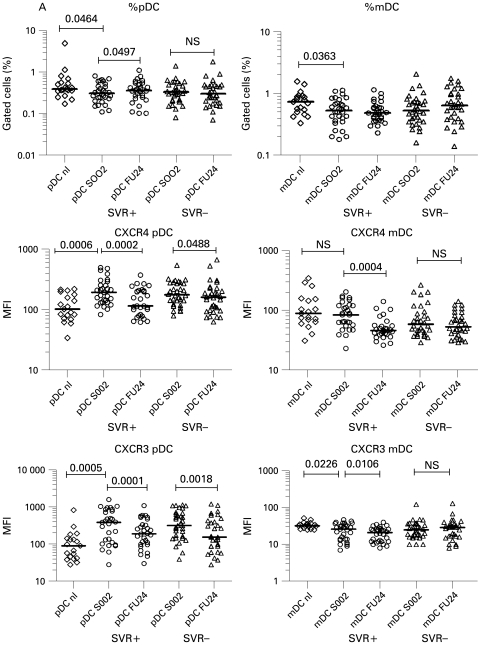
Differential elevations of chemokine receptors and maturation markers in dendritic cell (DC) subtypes before and after therapy. SOO2 is screening visit 2, 2 weeks before initiation of therapy. FU24 is 24 weeks after cessation of treatment. Median fluorescence intensity (MFI) values for each patient are shown. The horizontal line is the median. A two-tailed non-parametric Wilcoxon signed rank test was used for paired data or the two-tailed Mann–Whitney test was used to calculate p values. (A) Per cent DCs; CXCR4 MFI for pDCs and mDCs; CXCR3 MFI for pDCs and mDCs. mDCs, myeloid dendritic cells; pDCs, plasmacytoid dendritic cells. (*Figure 3** continues on p 970.*)

Pre-treatment migration of pDCs to CXCL12 was higher in SVR− patients (median 6.0, range 1.3–12) compared to SVR+ patients (median 3.8, range 1.4–5.4) (p = 0.0185, fig 4). Pre-treatment migration of pDCs to CXCL10 was also slightly higher in SVR− patients (median 1.29, range 0.69–1.7) vs (0.78 0.56–1.06, p = 0.0106, fig 4). Chemotaxis decreased after therapy but this change was not statistically significant. Myeloid DCs from SVR+ and SVR− patients showed no significant differences in their responses to CXCL12 or CXCL10 but the trends in migration were similar to pDCs (data not shown). Increasing pDC migration to CXCL12 correlated significantly with pre-treatment total HAI score (18 maximum points)^37^ ^48^ and periportal+portal inflammation subscores (8 maximum points) (p = 0.0329, p = 0.0234, respectively; fig 5), but not lobular inflammation. There was no association between chemotaxis and viral level (data not shown). Migration of pDCs to CXCL10 directly correlated with periportal+portal inflammation (p = 0.0399) but only approached statistical significance for total HAI score (p = 0.0640; fig 5). In contrast, mDC migrations to CXCL12 and CXCL10 were not significantly associated with inflammation (data not shown).

**Figure 3 Continued:**
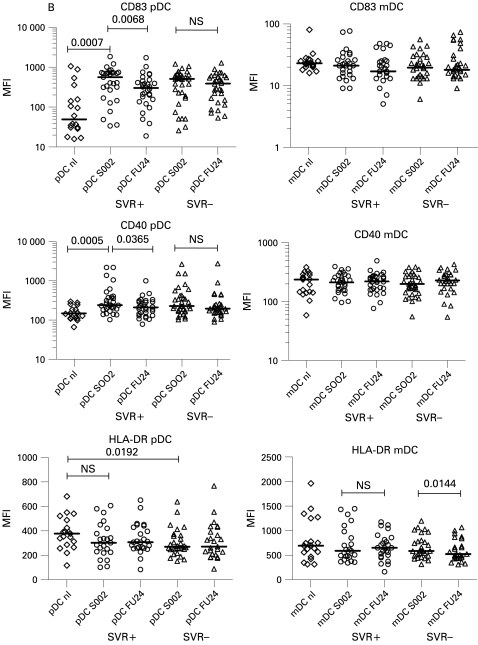
Differential elevations of chemokine receptors and maturation markers in dendritic cell (DC) subtypes before and after therapy. SOO2 is screening visit 2, 2 weeks before initiation of therapy. FU24 is 24 weeks after cessation of treatment. Median fluorescence intensity (MFI) values for each patient are shown. The horizontal line is the median. A two-tailed non-parametric Wilcoxon signed rank test was used for paired data or the two-tailed Mann–Whitney test was used to calculate p values. (B) CD83 MFI on pDCs, mDCs; CD40 MFI for pDCs, mDCs; HLA-DR for pDCs and mDCs.

**Figure 4:**
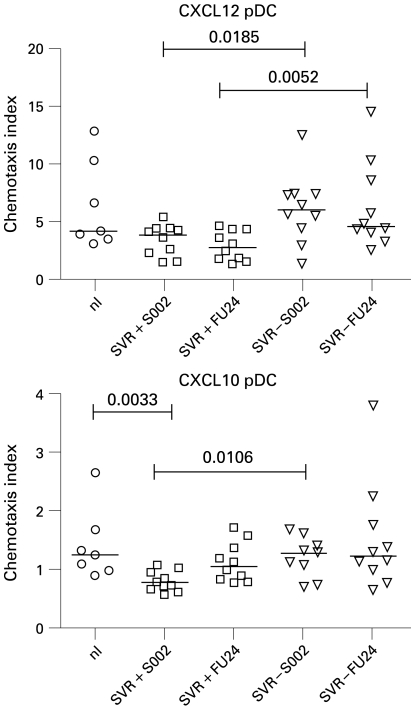
Increased baseline plamacytoid dendritic cell (pDC) chemotaxis to CXCL12, CXCL10 in SVR− patients. Ten SVR+ and 10 SVR− patients from the original cohort of 64 patients were compared with seven normal controls. Chemotaxis index (CI) was defined as the number of cells migrated with chemokine divided by the number of cells that migrated without chemokine. CXCL12 was used at 200 ng/ml, CXCL10 at 1 μg/ml. The Mann–Whitney test or the Wilcoxon signed rank test (for paired samples) was used to calculate p values. The horizontal line is the median. (A) Chemotaxis of pDCs to CXCL12. (B) Chemotaxis of pDCs to CXCL10 (note: 9/10 patients studied in the SVR− group had sufficient PBMCs for study).

## DISCUSSION

HCV may inhibit the immune response of DCs, hindering the adaptive response from T cells.^13^ ^47^ We hypothesised that DC dysfunction may determine the response to PEG-IFN/ribavirin therapy. In this large HCV treatment study of 64 patients, using ex vivo analysis, we found that pDCs and mDCs were decreased compared to normal controls, consistent with prior studies in HCV and similar to prior studies of patients with HIV.^13^ ^18^ ^19^ ^38^ ^44^ ^45^ ^49^ For the first time, we found that pDCs from HCV patients had elevated levels of the inflammatory chemokine receptors CXCR4 and CXCR3 before therapy, and, importantly, successful therapy was associated with larger decreases in expression levels. High chemokine and inflammatory cytokine levels in the blood of HCV patients likely increases chemokine receptor expression.^32^ The co-stimulatory molecule CD40 and the maturation marker CD83 were elevated on pDCs and decreased with SVR. Lower pre-treatment HLA-DR was seen on pDCs in HCV patients compared to normal controls. The maturation marker DC-SIGN, and the co-stimulatory molecule CD86 were not elevated, suggesting that pDCs in the blood had not achieved full maturation. Individual baseline DC marker phenotype did not predict SVR, including data analysis by race comparing Caucasians versus African–Americans.

For mDCs, there were fewer differences in marker expression between HCV and normal patients and the differences in expression level were small, suggesting that HCV infection affects pDCs in the peripheral blood more than mDCs. Supporting our findings, a recent paper demonstrated that infectious cell culture-derived HCV inhibited pDC IFNα production whereas mDCs were not affected.^47^

**Figure 5:**
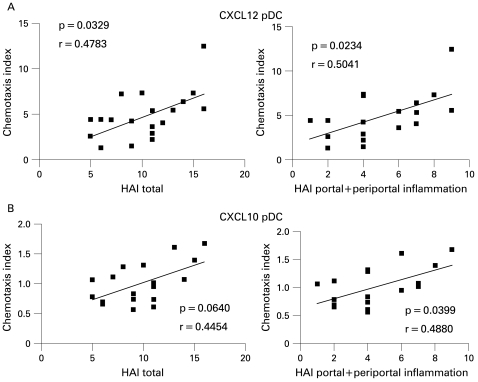
Increased plasmacytoid dendritic cell (pDC) chemotaxis to CXCL12, CXCL10 correlates with inflammation. Combining all 20 patients together, chemotaxis index at S002 (pre-treatment) was plotted against the total histological activity index (HAI)^37^ ^48^ (total 0–18) which includes a sub-score for periportal inflammation, portal inflammation, and fibrosis from the patients’ liver biopsy. The p value and correlation coefficient R for the association between the variables is shown. Analysis performed by GraphPad Prism software using the two-tailed Spearman’s test. (A) CXCL12 pDC total HAI score, CXCL12 periportal+portal HAI score. (B) CXCL10 pDC total HAI score, CXCL10 pDC periportal+portal HAI score.

Fully activated DCs express the chemokine receptor CCR7 which is important for lymph node homing, HL-DR for antigen presentation, co-stimulatory and maturation markers like CD40, CD86 and CD83.^14^ Partial or incomplete activation seen in our study has also been described in DCs from other chronic viral infections, such as HIV.^38^ A recent study of HIV patients showed elevated CD40 on mDCs and pDCs with low CD86, with even higher levels of CD40 seen in lymph nodes.^38^ In chronic HCV, this phenotype may result from chronic infection and inflammation in the liver and peripheral blood that is not sufficiently robust enough to cause full activation. These partially activated pDCs may be poor presenters of antigen because of low MHC surface levels and poor activators of T cells because they have low levels of co-stimulatory molecules. However, we recognise a limitation of our study was that we sampled DCs from the peripheral blood, a compartment where they are transiently; thus, we might be observing partial activation occurring secondary to inflammatory cytokine stimulation before DC migration to areas of inflammation.

We chose to study chemotaxis of DCs after observing differences in chemokine receptor levels between treatment response groups and because a prior study^30^ suggested that chemotaxis may be altered in HCV infection. Natterman *et al* described increased pDCs and mDCs in the livers of chronic HCV patients that may explain the decrease of DC seen in the periphery.^30^ Additionally, sera from HCV patients, and the HCV protein E2 inhibited in vitro migration to CCL21, a CCR7-binding chemokine important for lymph node homing. Natterman *et al* hypothesised that DCs are trapped in the liver and are unable to activate T cells in lymph nodes. We feel that our data are consistent with their hypothesis. We found that baseline chemotaxis to the inflammatory chemokines CXCL12 and CXCL10 was elevated in all patient groups compared with normal controls, consistent with our chemokine receptor expression data. Additionally, SVR− patients demonstrated increased pre-treatment chemotaxis to CXCL12 and CXCL10 compared to SVR+ patients. Hepatic inflammation levels showed significant correlation with chemotaxis in the patients studied. These observations are in accord with prior studies demonstrating that higher plasma pre-treatment levels of CXCL10 and CXCL8 were associated with an unfavourable response to combination therapy.^33^^–^35 A limitation of our study was that we were unable to study intrahepatic DCs from our patients to determine whether DC egress to the lymph node is compromised as proposed by Natterman *et al*.^30^

Why is increased baseline inflammation associated with a poorer response to therapy? Recent studies of gene expression in liver biopsy samples and PBMCs have demonstrated that patients with higher levels of interferon-stimulated genes before therapy have a poorer response to interferon/ribavirin.^50^^–^52 These results suggest that non-responding patients already have maximal interferon responses and that adding additional interferon does not augment the antiviral immune response.

In summary, pDCs from patients with chronic HCV analysed directly ex vivo show increased inflammatory chemokine receptor expression and a partial activation phenotype that suggests pDC dysfunction. Successful antiviral therapy normalises many phenotypic and functional abnormalities of pDCs from patients with HCV. Our finding that pDCs in SVR− patients demonstrate increased chemotaxis before therapy reinforces the hypothesis that increased baseline inflammation is associated with failure to respond to antiviral therapy. It remains to be determined if DC migration from liver to lymph node and/or antigen presentation to T cells is inhibited in vivo. Further functional studies of HCV DCs may lead to improved immune-boosting therapies for viral eradication.
